# Semi-Supervised Adversarial Learning Framework for Controller Area Network Bus Intrusion Detection †

**DOI:** 10.3390/s26123964

**Published:** 2026-06-22

**Authors:** Jonggwon Kim, Hyungchul Im, Semin Kim, Seongsoo Lee

**Affiliations:** Department of Intelligent Semiconductors, Soongsil University, Seoul 06978, Republic of Korea; jonggwon@soongsil.ac.kr (J.K.); tory@soongsil.ac.kr (H.I.); semin2371@soongsil.ac.kr (S.K.)

**Keywords:** automotive cybersecurity, sensor cybersecurity, controller area network, intrusion detection system, generative adversarial network, semi-supervised learning, vehicular sensing

## Abstract

Modern connected vehicles rely on the controller area network (CAN) to disseminate safety-critical in-vehicle information, including sensor-related and vehicle-state signals such as engine revolutions per minute (RPM) and gear state, among electronic control units (ECUs). Because CANs lack built-in authentication and encryption, malicious message injection and spoofing can compromise the integrity and availability of vehicular sensing and control functions. Existing deep-learning-based intrusion-detection systems (IDSs) show a clear trade-off: supervised methods perform well on known attacks but rely on costly labels, whereas unsupervised methods can identify unseen attacks but often suffer from high false-positive rates. To address these limitations, this paper proposes a semi-supervised generative adversarial network (SGAN) framework for CAN bus intrusion detection that combines image-based CAN representation with adversarial learning. Consecutive CAN messages are converted into 64×9 grayscale images, and the proposed framework is trained in three phases. First, the discriminator establishes an initial decision boundary using a small labeled subset. It then refines this boundary through distribution-level likelihood objectives and generated samples. Finally, the generator is trained to produce realistic samples capable of deceiving the discriminator. The proposed method was evaluated on the Hacking and Countermeasure Research Lab (HCRL) car-hacking dataset using leave-one-class-out experiments to simulate unknown attacks and achieved an average accuracy of 99.73% and an average F1-score of 99.63% on unknown attacks. Moreover, with only 0.21 M parameters and 3.25 M floating-point operations (FLOPs), the model is well suited for resource-constrained in-vehicle platforms. These results indicate that the proposed framework can serve as a practical cybersecurity component for protecting CAN-carried data in vehicular sensing applications.

## 1. Introduction

The automotive industry is shifting from mechano-centric structures to highly integrated electronic control systems, driven by advancements in autonomous driving and connected car technologies. This functional diversification has led to a sharp increase in the number of in-vehicle electronic control units (ECUs), significantly compounding system complexity [1]. Furthermore, as vehicle-to-everything (V2X) communication enables real-time connectivity with the external infrastructure and other vehicles, the automobile has evolved from a conventional mode of transport into a sophisticated intelligent computing platform [2]. Although these advancements offer unprecedented convenience, the expanded connectivity is exposing previously isolated internal networks to direct cyber threats [3,4].

Modern vehicles utilize a variety of networks, including the local interconnect network (LIN), FlexRay, media-oriented systems transport (MOST), automotive Ethernet, and the controller area network (CAN) [5]. Within this heterogeneous environment, the CAN remains the dominant protocol for critical control domains such as engine and braking systems, owing to its high reliability and real-time performance [6]. However, the CAN possesses inherent structural vulnerabilities, notably the absence of authentication to verify message origins and lack of encryption for data protection. Specifically, the broadcast-based nature of the CAN, which delivers messages to all connected nodes, presents a critical security flaw. Once an external attacker gains network access, it can inject an unlimited number of malicious messages to hijack vehicle functions, which can paralyze the entire system [7].

To counter these security threats, extensive research has focused on intrusion-detection systems (IDSs) that analyze network traffic in real time to detect malicious behavior. Early studies primarily utilized rule-based or statistical methodologies [8]. However, these approaches struggled to identify sophisticated attack patterns, leading to the recent prominence of deep-learning-based IDSs [9]. These systems are generally categorized into supervised and unsupervised learning depending on the availability of training labels. Supervised learning models are trained on datasets labeled with both normal and attack data. Prominent examples include deep convolutional neural networks (DCNNs), which utilize convolutional neural network (CNN) architecture to extract spatial features [10], and long short-term memory (LSTM), which is designed for processing time-series data [11]. Although these models ensure high detection accuracy for known attack patterns, they possess a fundamental limitation: limited ability to detect unknown attacks or those that mimic normal traffic. Furthermore, they incur significant computational and manual costs when labeling vast amounts of data for model training. By contrast, unsupervised learning involves models that learn the inherent characteristics or probability distributions of the input data without explicit labels. A representative example is the one-class support vector machine (OCSVM), which identifies abnormal traffic by learning the periodic characteristics of normal messages [12]. Unsupervised learning methods have the advantage of requiring no explicit labeling and can respond flexibly to unknown attacks. However, because of the absence of clear decision boundaries, they tend to have lower detection precision than supervised learning, which significantly compromises the overall reliability of the system.

In this paper, we propose a semi-supervised generative adversarial network (SGAN) framework for CAN intrusion detection that addresses the trade-off between supervised accuracy and unsupervised flexibility. The proposed system converts consecutive CAN messages into compact grayscale images so that a CNN-based discriminator can capture structural patterns in the message stream. Its key methodological contribution is a three-phase strategy that combines sparse-label decision boundary initialization, distribution-level likelihood refinement, and adversarial sample generation. By integrating these components, the framework aims to improve robustness to unknown attacks while keeping labeling requirements low.

This manuscript extends our conference paper on an SGAN-based IDS for in-vehicle networks [13]. Compared with the conference version, the present manuscript clarifies the preprocessing framework so that the same normalization scheme supports both CAN 2.0A and CAN 2.0B. It also provides a more detailed description of the three-phase SGAN training and inference procedure. In addition, it expands the discussion of the evaluation protocol, limitations, and deployment considerations.

From the perspective of vehicular sensing, the CAN bus in modern vehicles is not merely a communication backbone but also a dissemination path for safety-critical sensor-related and vehicle-state information, such as engine revolutions per minute (RPM) and gear state, exchanged among ECUs. Therefore, attacks on CAN traffic directly threaten the integrity, availability, and trustworthy use of data that support sensor-enabled automotive functions. Accordingly, this study addresses CAN intrusion detection as a sensor-data integrity problem and investigates the proposed SGAN as a cybersecurity mechanism for secure and reliable vehicular sensing applications.

The major contributions of this paper are as follows:

1. We present a CAN intrusion detection framework with low labeling cost that establishes an initial decision boundary using only 5% labeled data and refines it through semi-supervised adversarial learning.
2. The proposed model employs a preprocessing scheme that converts consecutive CAN messages into fixed-size grayscale images. By normalizing message identifiers (IDs), the method supports both CAN 2.0A and CAN 2.0B.
3. We validate the proposed framework under leave-one-class-out evaluation and show that it achieves an average unknown attack accuracy of 99.73% and an average unknown attack F1-score of 99.63%. Moreover, the proposed model requires only 0.21 M parameters and 3.25 M floating-point operations (FLOPs).

The remainder of this paper is organized as follows: Section 2 reviews the related work, and Section 3 provides the necessary research background. Section 4 presents a detailed description of data preprocessing, network architecture, and the proposed learning methodology. Section 5 discusses the experimental results through a comprehensive analysis and addresses the limitations of the research. Finally, Section 6 concludes the paper.

## 2. Related Work

To complement the performance of conventional statistical or rule-based detection models, research leveraging deep learning has become prevalent in analyzing the nonlinear patterns of CAN traffic. Supervised learning studies focus on maximizing performance by training on labeled datasets to capture the inherent spatiotemporal features of CAN traffic. Song et al. [10] proposed a DCNN model that captures the spatial correlations between data elements by transforming CAN message bit values into two-dimensional grids. Hossain et al. [11] demonstrated high detection performance against denial of service (DoS) and spoofing attacks using an LSTM network that captures time-series characteristics. Desta et al. [14] proposed Rec-CNN, which encodes intricate message characteristics by applying recurrence plots to visualize nonlinear patterns in time-series data. Gao et al. [15] introduced CanNet, a lightweight image-based architecture tailored to the resource-constrained environment of in-vehicle-embedded devices. This model confirms the feasibility of supervised IDS in real-vehicle environments by maintaining high accuracy with minimal computational overhead. More recent deep-learning-based studies have further extended this direction toward protocol compatibility and lightweight deployment. Aung et al. [16] proposed CANDIDS, a deep-learning-based IDS designed to operate on both conventional CAN and CAN with flexible data rate (CAN FD) traffic. Zhao et al. [17] proposed a lightweight CAN bus IDS based on a depthwise separable convolutional Kolmogorov–Arnold network, aiming to balance detection accuracy and model complexity in resource-constrained environments. Although supervised models excel in countering predefined attack types, they remain vulnerable to unknown attacks in real-world scenarios. Furthermore, they face a fundamental challenge regarding the prohibitive costs of labeling the vast datasets required to keep pace with increasingly sophisticated attack scenarios.

Accordingly, unsupervised-learning-based research, which identifies attack patterns by learning only the distribution of normal data, has emerged as a promising alternative. Taylor et al. [12] proposed an OCSVM-based method that establishes decision boundaries based on the transmission frequency and time intervals of normal traffic. Within this framework, any patterns deviating from established boundaries are identified as attacks. Longari et al. [18] proposed CANnolo, which utilizes an LSTM autoencoder to capture the periodicity and sequential characteristics of CAN data. By analyzing CAN ID sequences within a sliding window, the model learns temporal dependencies in the process of being optimized to reconstruct normal traffic patterns. Consequently, it leverages the high reconstruction errors induced by attack message injections as primary indicators of anomaly detection. Seo et al. [19] proposed a generative adversarial network (GAN)-based intrusion-detection system (GIDS) that applies a GAN to in-vehicle network security. This model employs a competitive learning process, in which a generator creates synthetic data mimicking normal distributions, and the discriminator learns to distinguish them from actual data. Through this process, the discriminator learns the latent patterns of the normal data, functioning as a sophisticated classifier that identifies abnormal deviations as attacks. Recent anomaly-detection studies have continued to explore unlabeled or normal-traffic-based modeling for CAN security. For example, Kim et al. [20] proposed an adaptive autoencoder-based CAN IDS that combines reconstruction-based anomaly detection with a kernel density estimation function, reflecting continued interest in unsupervised detection without extensive attack labels. However, because unsupervised models are trained without explicit attack labels, they may have difficulty establishing clear decision boundaries between normal and malicious traffic. Reconstruction-based approaches, in particular, rely heavily on reconstruction error as the primary detection criterion. Poorly calibrated anomaly thresholds can therefore increase false positives or cause attacks resembling normal traffic to be overlooked, thereby reducing overall reliability.

Semi-supervised learning, which integrates supervised and unsupervised techniques to refine detection performance, has emerged as a promising alternative in addressing the limitations of unsupervised decision boundaries. In a representative study, Hoang et al. [21] proposed a convolutional adversarial autoencoder (CAAE) that applies adversarial learning to an autoencoder framework. It constructs a sharper decision boundary for the normal category by training not only on normal data but also on unlabeled attack data as unknown samples. Nguyen et al. [22] proposed a model that combines a variational autoencoder (VAE) with adversarial environment reinforcement learning (AERL). This model first pre-trains the entire unlabeled dataset using a VAE. Subsequently, it trains a teacher model with 50% of the total data and artificially expands the training dataset by assigning pseudo-labels to the remaining unlabeled data. More recently, Huang et al. [23] proposed FSL-IDS, a federated semi-supervised intrusion-detection system for in-vehicle networks, indicating that recent semi-supervised research is also moving toward distributed low-label learning and resource-aware deployment. However, the CAAE and VAE-AERL approaches described above use unlabeled or pseudo-labeled attack samples during training. Therefore, their evaluations do not fully demonstrate detection of attack classes that are completely absent from the training process. Moreover, assigning pseudo-labels to unlabeled data may propagate classification errors when unseen attacks differ substantially from known patterns. To address these limitations, the proposed SGAN combines the explicit decision boundary learning of supervised methods with the distribution modeling capability of unsupervised adversarial learning. This design aims to retain the high classification accuracy of supervised learning while improving robustness to previously unseen attacks with only a small amount of labeled data.

## 3. Background

### 3.1. Controller Area Network

The CAN is currently the dominant international standard for in-vehicle communication [24]. It enables numerous ECUs, such as engine, braking, and steering controllers, to exchange data over a shared bus without requiring a complex central computer. This approach reduces the overall vehicle weight by minimizing the wiring and ensures high reliability, enabling robust communication even in electrically noisy driving environments.

**Figure 1:**
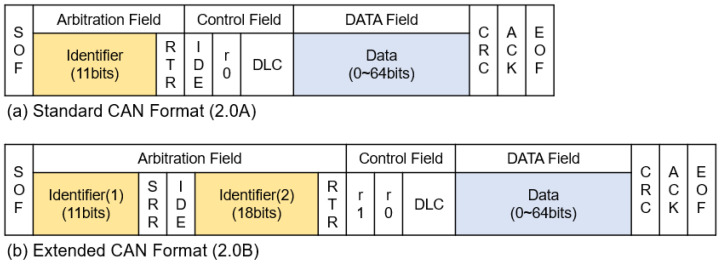
Structure of CAN frames: (**a**) standard CAN 2.0A frame and (**b**) extended CAN 2.0B frame.

Figure 1 illustrates the detailed structure of a CAN frame, which is primarily characterized by an ID-based broadcast mechanism rather than an address-based approach. A unique ID is used instead of source and destination addresses. All receiving nodes observe every network message. Each node then selectively processes only the messages whose IDs appear in its acceptance list. The CAN protocol is categorized into two specifications based on the identifier length. Figure 1a shows the standard protocol, which uses an 11-bit ID to determine message priority. The arbitration field consists of this 11-bit ID and a remote transmission request (RTR) bit, which indicates whether it is a data frame. By contrast, the extended protocol shown in Figure 1b provides a 29-bit ID by appending an 18-bit extended ID to the base 11-bit ID. This extension accommodates the growing demand for unique IDs driven by increasing system complexity. The two protocols are distinguished by the value of the identifier extension (IDE) bit in the arbitration field.

**Figure 2:**
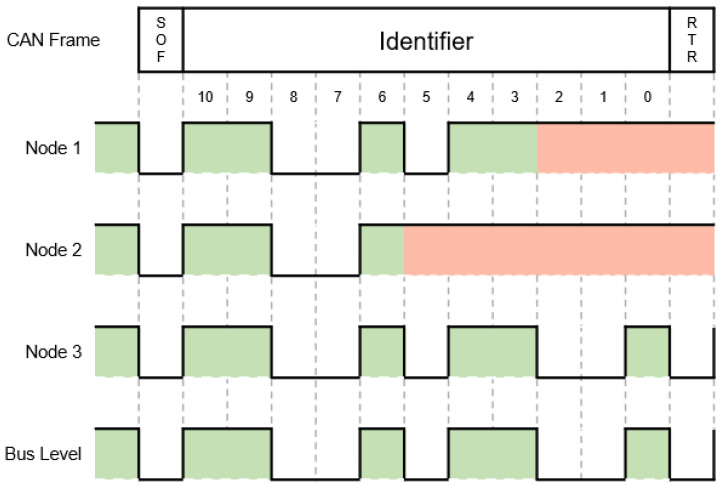
Principle of CAN bus arbitration among nodes 1–3. The numbers identify the transmitting nodes and bit positions.

Figure 2 illustrates the bitwise arbitration process, which is the CAN mechanism for resolving collisions that occur when multiple frames are transmitted simultaneously. Unlike conventional networks, any node in a CAN system can attempt to access the network at any time. If two or more nodes initiate a transmission simultaneously, priority is determined using the previously described ID field. The CAN bus follows a wired-AND logic structure in which the dominant value “0” electrically overrides the recessive value “1”. For example, at bit 5, node 2 transmits a recessive “1” but the bus maintains a dominant “0” state due to the signals from nodes 1 and 3. Subsequently, at bit 2, node 1 transmits a recessive “1” while node 3 sends a dominant “0” that forces the bus to remain “0”. Consequently, node 3 secures bus access, whereas nodes 1 and 2 halt transmission and switch to receive mode. Through this mechanism, CAN ensures that high-priority messages are delivered immediately without data loss from collisions or retransmission delays [25].

### 3.2. Generative Adversarial Networks

A GAN is an unsupervised learning framework consisting of two networks, a generator and discriminator, working in opposition to each other to learn the latent probability distribution of the data [26]. The training process of this model follows the minimax game principle of game theory. It is based on a zero-sum structure in which two networks compete with opposing objective functions to reach an optimal equilibrium.

Specifically, the generator aims to deceive the discriminator by synthesizing fake data that are statistically indistinguishable from the actual data based on random noise vectors received from latent space. By contrast, the discriminator aims to maximize the accuracy of authenticity determination by performing binary classification to determine whether the input belongs to an actual training dataset or is a synthetic sample. In other words, both networks update their parameters through mutual interaction. The generator attempts to induce misclassification by the discriminator. In response, the discriminator learns to distinguish the deception introduced by the generator.

As this competitive learning is repeated, the generator acquires the ability to mimic the sophisticated features of normal data. Ideally, the model reaches the Nash equilibrium, with the distribution of the generated data, $p_{g}$, perfectly aligning with the actual data distribution. At this point, the discriminator converges to a probabilistic limit of 0.5, implying that it can no longer distinguish between real and synthetic inputs. Consequently, the trained discriminator internalizes the underlying distribution of normal traffic, providing a robust mechanism for identifying abnormal patterns that deviate from the established normal domain.

### 3.3. Semi-Supervised Learning

Semi-supervised learning is a framework that trains a model using a small amount of labeled data and a large volume of unlabeled data [27]. It is designed to mitigate the prohibitive costs of data labeling in supervised learning while reducing the performance uncertainty inherent in unsupervised learning. In general, supervised learning ensures a high classification performance by training on ground truth labels. However, it is limited by the significant costs and time required to collect high-quality labeled data and remains vulnerable to unknown attacks that are not present in the training dataset. By contrast, unsupervised learning enhances data collection efficiency by learning inherent patterns without labels; however, it often suffers from lower precision because of the absence of clear classification guidelines.

Semi-supervised learning has emerged as an alternative that overcomes these limitations by leveraging the strengths of both approaches. This technique establishes an initial decision boundary using a small set of labeled data. It then iteratively refines this boundary by learning the structural features of the entire dataset from a large volume of unlabeled data. Consequently, it maximizes performance by balancing supervised classification accuracy with unsupervised data scalability.

The utility of semi-supervised learning is particularly notable in the field of in-vehicle network security. In real-world driving environments, although normal CAN messages are readily available, securing actual attack scenarios or labeled data is extremely challenging [9]. Semi-supervised learning addresses this imbalance by using sparsely labeled data as the primary identification criteria while leveraging vast amounts of unlabeled data to learn the underlying structural distribution. This approach is highly effective for maximizing both detection precision and data efficiency while providing comprehensive coverage across a wide range of driving scenarios [28]. In the proposed SGAN framework, this semi-supervision is realized by using sparse explicit labels only in Phase 1 and by refining the decision boundary in later phases through likelihood-based objectives and adversarially generated samples.

## 4. Proposed Method

### 4.1. Overall Framework

**Figure 3:**
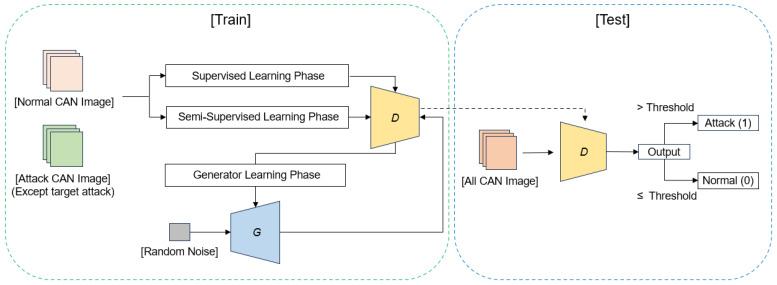
Overall framework of the proposed CAN IDS. G, generator; D, discriminator.

The proposed SGAN-based IDS consists of three stages: data preprocessing, model training, and anomaly detection. The overall system architecture and data flow are presented in Figure 3. Raw messages in time-series format collected from the in-vehicle CAN bus are fed into the preprocessing module. In this module, the CAN IDs and data fields are normalized and converted into two-dimensional grayscale images. This transformation represents the message stream in a spatial form that is well suited to CNN-based feature extraction. In the subsequent training phase, an SGAN framework consisting of a generator and a discriminator is implemented. The generator creates fake images from noise in latent space. Receiving both actual and synthetic images, the discriminator performs binary classification training to determine whether an attack has occurred. During training, the discriminator first uses a small labeled subset to establish an initial decision boundary. It is then refined with additional training data and generated fake data through likelihood-based objectives that shape the normal and abnormal regions in the output space. In this way, the model sharpens the normal boundary without introducing a separate pseudo-labeling step. Finally, the trained discriminator calculates an anomaly score for each input CAN image. Because the discriminator output is a sigmoid score in [0,1] and the attack and normal classes are encoded as 1 and 0, respectively, we use 0.5 as a predefined midpoint decision threshold. The same threshold is applied to all scenarios without attack-specific or test set tuning. Scores above 0.5 are classified as attacks; otherwise, the corresponding CAN frame sequence is classified as normal.

### 4.2. Preprocessing Method

As CNNs are specialized in learning the spatial patterns of input data [29], this paper proposes a preprocessing technique that converts a time-series CAN message stream into a 64×9 two-dimensional grayscale image. In this process, only the CAN ID and data fields that determine the unique characteristics of the message are extracted. This approach maximizes computational efficiency. A grayscale encoding was adopted because each selected CAN attribute is inherently a scalar quantity: the normalized CAN ID and the eight payload bytes. Representing these values with a single intensity channel preserves their native numeric semantics without introducing artificial color-channel structure. In addition, grayscale inputs reduce memory traffic and convolutional cost relative to multi-channel images, which is desirable for ECU-oriented deployment. The 8-bit intensity range is also naturally compatible with the 0–255 value range of CAN data bytes, enabling direct encoding of payload bytes and compact normalization of CAN IDs. The entire preprocessing procedure consists of two stages: single-frame encoding and image generation.

In single-frame encoding, each CAN frame is converted into a 1×9 pixel vector. The first element of the vector represents the CAN ID, and the remaining eight elements represent each byte in the data field. Because the range of a CAN ID varies according to the protocol version, normalization is performed to map it to an 8-bit grayscale range of 0 to 255. Let the CAN ID at time *t* be ${\mathrm{ID}}_{t}$. The converted pixel value $p_{\mathrm{ID}}$ is calculated as follows:

(1) $$p_{\mathrm{ID}}=\left\lfloor \frac{{\mathrm{ID}}_{t}}{{\mathrm{ID}}_{\max}}\times 255\right\rfloor$$

where ${\mathrm{ID}}_{\max}$ is the maximum value determined by the identifier bit-length of the CAN protocol. For CAN 2.0A (11-bit ID), ${\mathrm{ID}}_{\max}$ is set to $2^{11}-1=2047$. Conversely, for CAN 2.0B (29-bit ID), it is set to $2^{29}-1$ = 536,870,911. That is, the proposed preprocessing method can process IDs from both standard and extended protocols by adjusting only ${\mathrm{ID}}_{\max}$. This allows the same preprocessing structure to support both standard and extended CAN formats without architectural changes. The 8-bit mapping is many-to-one: neighboring IDs, including those near the lower and upper bounds, can share the same grayscale value, with average quantization intervals of approximately 8 IDs for CAN 2.0A and $2.1\times 10^{6}$ IDs for CAN 2.0B. This compression may reduce ID-level discrimination, particularly for CAN 2.0B, although the payload bytes and temporal patterns provide complementary information. For the data field, both CAN 2.0A and CAN 2.0B share the same specification of a maximum of 8 bytes. Because each byte $D_{i}$ already has a value within the range of 0–255, it is mapped directly without additional scaling. Furthermore, data payloads smaller than 8 bytes are padded with 0 to maintain consistent dimensions. This keeps the input format fixed across protocol versions in both ID and data field processing.

**Figure 4:**
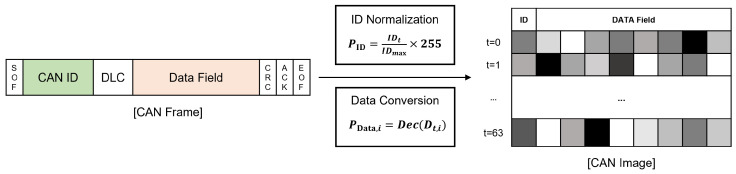
The proposed data preprocessing framework.

During image generation, the temporal correlation between consecutive CAN messages is represented as a spatial feature. To achieve this, the converted 1×9-pixel vectors are stacked vertically to generate a two-dimensional image. In this paper, the window size was set to 64, organizing 64 consecutive CAN frames into a single 64×9 grayscale image. If at least one attack frame was included within these 64 frames, the generated image was labeled as an attack. As shown in Figure 4, the generated image represents time along the vertical axis and message attributes along the horizontal axis.

### 4.3. Network Architecture

The proposed SGAN model was designed based on a deep convolutional generative adversarial network (DCGAN) architecture that applies asymmetric kernels to effectively process the 64×9 rectangular images. Specifically, the kernel size and stride are optimized by considering the asymmetric data characteristics of the width and height ratios. The detailed architectures of the generator and discriminator are presented in Table 1.

The generator receives a 256-dimensional latent vector as input and transforms it into a 64×9 fake image, $x_{\mathrm{fake}}$, that matches the dimensions of the actual CAN image. It consists of five transposed convolutional layers that progressively upsample low-dimensional feature maps. Starting with a (4,1) kernel in the initial layer, asymmetric kernels, such as (4,2) and (4,3), are applied stepwise to expand the 1×1 input to a final resolution of 64×9. To ensure training stability, the rectified linear unit (ReLU) activation function is applied to all layers, except for the final output layer, and batch normalization is applied to certain layers. For the final output layer, a tanh function is used to normalize the output values to the range of [−1,1].

The discriminator is a binary classifier that determines whether an input image of size 64×9 is normal or an attack. Instead of using pooling layers, the discriminator extracts features while reducing the spatial dimensions through strided convolutions. The discriminator design comprises five convolutional layers to maintain structural symmetry with the generator, using asymmetric kernels and strides such as (4,3), (4,2), and (4,1) to compress the image into a scalar value. This allows the model to effectively capture distinct information from the time-series axis (height) and feature axis (width). After applying the ReLU activation function to all hidden layers, the final flattened feature vector is passed through a sigmoid function to output the probability of an attack as a value between 0 and 1.

### 4.4. Three-Phase Training Methodology

The proposed framework is based on a stepwise training strategy. First, an initial decision boundary is established using explicit labels for only 5% of the total data. The boundary is then refined with an additional 25% training subset in Phase 2, and the remaining data are reserved for out-of-sample evaluation. This split was selected to emulate a practical low-label automotive IDS setting in which only a small portion of the traffic can be carefully annotated, while a larger pool of data is available for subsequent boundary refinement. The framework is semi-supervised in the overall sense. Explicit supervised learning is restricted to the small labeled subset in Phase 1. The later phases refine the boundary through distribution-level objectives and adversarially generated samples instead of assigning new hard labels to all remaining data. These ratios should therefore be interpreted as a practically motivated operating point rather than as universally optimal values. The 5%/25% split was fixed before evaluation and applied identically to all leave-one-class-out scenarios. It was not selected by optimizing the reported metrics, and the target unknown attack class was excluded from both the labeled subset and the Phase 2 training subset in each scenario. This process consists of three distinct stages: initial boundary formation, boundary refinement, and generator optimization.

- Phase 1: In the first phase, the discriminator learns the initial decision boundary between normal and attack data using a small amount of labeled data, which constitutes 5% of the total dataset. The primary objective of this phase is to enable the model to capture the fundamental features of the data. The discriminator is trained to output 0 for actual normal data $x_{\mathrm{normal}}$ and 1 for actual attack data $x_{\mathrm{attack}}$. The supervised loss function $L_{\sup}$ is defined as the expected value of the binary cross entropy between the input *x* and ground truth label *y*, derived from the labeled data distribution $p_{\mathrm{labeled}}$.
  (2) $$L_{\sup}=-E_{x,y}\left[y\log D(x)+(1-y)\log(1-D(x))\right]$$
  where D(x) represents the output probability of the discriminator for the input *x*, and *y* denotes the ground truth label. This equation induces the model to generalize and learn the conditional probability distribution inherent in the labeled data, rather than being biased toward specific samples. The discriminator generates initial weights for distinguishing between normal and attack samples by updating its parameters in a direction that minimizes this expected value. For example, if the discriminator predicts a normal sample as a value close to 1 or an attack sample as a value close to 0, the negative log value increases significantly. This assigns a larger penalty to the model, compelling it to update the weights to correct the misclassification. Training proceeds in a direction that minimizes the expected loss over the entire data distribution, thereby establishing a decision boundary that clearly distinguishes between normal and attack samples.
- Phase 2: In the second phase, the discriminator is refined with an additional training subset (25% of the total data) and the fake data created by the generator. This phase is not a pseudo-labeling step. Instead, it updates the boundary learned in Phase 1 through distribution-level log-likelihood objectives that shape the normal and abnormal regions in the discriminator output space. The total loss function $L_{\mathrm{semi}}$ is defined as the sum of the expected values for each distribution as follows:
  (3) $$L_{\mathrm{semi}}=L_{\mathrm{normal}}+L_{\mathrm{attack}}+L_{\mathrm{fake}}$$
  where $L_{\mathrm{normal}}$, $L_{\mathrm{attack}}$, and $L_{\mathrm{fake}}$ denote the expected losses for normal samples, known attack samples available during training, and generator-produced fake samples, respectively. First, the specific loss term $L_{\mathrm{normal}}$ for the normal data distribution $p_{\mathrm{normal}}$ is expressed as follows:
  (4) $$L_{\mathrm{normal}}=-E_{x∼p_{\mathrm{normal}}}\left[\log(1-D(x))\right]$$
  where $L_{\mathrm{normal}}$ encourages the discriminator to map normal data toward 0. If normal data are pushed toward the attack side, the loss increases sharply, forcing the model to preserve a compact normal region. Next, the loss terms for the attack data distribution $p_{\mathrm{attack}}$ and fake data distribution $p_{z}$ are defined as follows, respectively:
  (5) $$L_{\mathrm{attack}}=-E_{x∼p_{\mathrm{attack}}}\left[\log D(x)\right]$$
  (6) $$L_{\mathrm{fake}}=-E_{z∼p_{z}}\left[\log D(G(z))\right]$$
  where *x* denotes a sample drawn from the Phase 2 training data, and *z* represents a noise vector randomly sampled from the latent space. Thus, $L_{\mathrm{attack}}$ is computed from known attack samples available during training, whereas $L_{\mathrm{fake}}$ is computed from the fake samples G(z) synthesized by the generator *G*. Together, these terms push known attack and generated samples toward 1. Consequently, Phase 2 should be understood as a likelihood-based boundary-refinement stage rather than a hard-label assignment stage. By pulling normal patterns toward 0 and pushing known attack and generated patterns toward 1, the discriminator tightens the normal region learned in Phase 1 and broadens the abnormal region around that boundary. This refined separation improves robustness to withheld attack classes at test time, which are detected as out-of-distribution patterns relative to the learned normal manifold.
- Phase 3: In the final phase, the generator is trained to synthesize realistic synthetic samples capable of deceiving the discriminator. The generator produces synthetic data G(z) based on a noise vector *z* from latent space, aiming to induce the discriminator to misclassify it as normal data. This represents a core process of adversarial competition that compels the discriminator to learn the normal-data distribution more accurately. The objective function of the generator, $L_{\mathrm{gen}}$, is defined as follows to maximize the probability that the discriminator classifies fake data G(z) as normal:
  (7) $$L_{\mathrm{gen}}=-E_{z∼p_{z}}\left[\log(1-D(G(z)))\right]$$
  where $L_{\mathrm{gen}}$ is optimized in a direction that maximizes the probability that the generated data G(z) are judged as normal by the discriminator, which is achieved by maximizing the expected value of (1−D(G(z))). If the discriminator accurately filters out the generated data as abnormal, the output probability D(G(z)) becomes 1 and the value inside the logarithm approaches 0. As a result, the overall loss value increases significantly, imposing a heavy penalty on the generator. This loss structure compels the distribution of the synthetic data to statistically align with the actual normal data distribution. Consequently, the generator imitates the latent features of normal traffic, and in response, the discriminator establishes a more precise boundary of normal data, thereby enhancing the overall anomaly detection performance.

## 5. Performance Evaluation

### 5.1. Experimental Datasets

In this paper, a car-hacking dataset from the Hacking and Countermeasure Research Lab (HCRL) was used to validate the performance of the proposed model [30]. This dataset was collected from the on-board diagnostics-II (OBD-II) port of a Hyundai YF Sonata (Hyundai Motor Company, Seoul, Republic of Korea). The entire dataset utilized in the experiment is summarized in Table 2 and comprises CAN messages generated during normal driving along with four types of malicious attacks: DoS, fuzzy, gear spoofing, and RPM spoofing. The detailed mechanisms and characteristics of each attack type are as follows:

- DoS Attack: The DoS attack violates the availability of the vehicle network by exploiting the arbitration mechanism of the CAN protocol. In CAN communication, a smaller message ID value indicates a higher priority. The attacker leverages this by setting the ID to 0x000, which has the highest priority, and injects messages with the data field filled with 0 into the bus at extremely short intervals. As a result, normal ECUs fail to gain access to the bus and enter a standby state. This can delay or disrupt in-vehicle communication.
- Fuzzy Attack: The fuzzy attack refers to a method of inducing malfunctions in the vehicle control system by injecting a large volume of randomly generated data without targeting a specific vulnerability. The attacker transmits CAN frames with arbitrary IDs and random data values into the bus at irregular intervals. These random messages cause the vehicle actuators to operate unintentionally when their IDs match those of normal control commands. Furthermore, they induce exception handling and system crashes by providing input values within a range that the ECU cannot process.
- gear Spoofing Attack: The spoofing attack exploits the absence of sender authentication in CAN messages, allowing an attacker to impersonate a normal ECU and transmit manipulated information. The gear spoofing attack involves injecting false gear information by masquerading as the transmission control unit. Modern safety mechanisms block physical gear shifts during high-speed driving. However, forged signals injected by an attacker occupy the bus at a higher frequency than the normal transmission control unit (TCU) broadcasts, disrupting the decision logic of receiving ECUs. This leads to system malfunctions, whereby electronic components dependent on the gear state are activated regardless of the actual driving conditions.
- RPM Spoofing Attack: The RPM spoofing attack targets the engine speed information transmitted by the engine control unit. The attacker injects forged messages containing RPM values unrelated to the actual engine state or close to 0. In this dataset, manipulated RPM signals are transmitted approximately every 1 ms. These signals cause the instrument cluster gauge to fluctuate abnormally and induce malfunctions in other engine-dependent ECUs, such as transmission timing control.

### 5.2. Experimental Setup

To quantitatively validate the intrusion detection performance of the SGAN, this paper employed the accuracy, precision, recall, and F1-score derived from a confusion matrix as evaluation metrics. In this experimental setup, attack data were defined as positive (1), whereas normal data were classified as negative (0). Based on these definitions, a true positive (TP) refers to cases in which actual attacks are accurately detected as attacks, whereas a true negative (TN) denotes cases in which normal data are correctly classified as normal. Conversely, false positives (FPs) represent false alarms where normal data are incorrectly judged as an attack, and false negatives (FNs) indicate missed detections where an actual attack is regarded as normal.

(8) $$\mathrm{Accuracy}\;\left(\%\right)=\frac{\mathrm{TP}+\mathrm{TN}}{\mathrm{TP}+\mathrm{TN}+\mathrm{FP}+\mathrm{FN}}\times 100$$

(9) $$\mathrm{Precision}\;\left(\%\right)=\frac{\mathrm{TP}}{\mathrm{TP}+\mathrm{FP}}\times 100$$

(10) $$\mathrm{Recall}\;\left(\%\right)=\frac{\mathrm{TP}}{\mathrm{TP}+\mathrm{FN}}\times 100$$

(11) $$F1\text{-}\mathrm{score}\;\left(\%\right)=\frac{2\times \mathrm{Precision}\times \mathrm{Recall}}{\mathrm{Precision}+\mathrm{Recall}}$$

Accuracy represents the proportion of TPs and TNs that are correctly classified by the model among all data samples. This is used as an intuitive measure to evaluate the overall discriminative capability of the system. However, precision and recall are essential in vehicle environments where data imbalance is frequent. Performance evaluation based on accuracy alone is insufficient in such contexts. Precision denotes the proportion of actual attacks among the data predicted as attacks by the model. Low precision leads to frequent false alarms during normal driving, which can cause unnecessary confusion for drivers. Recall indicates the proportion of actual attack data successfully detected by the model. Low recall implies the occurrence of missed detections where actual threats are not identified, which can lead to fatal consequences, such as loss of vehicle control or accidents. Finally, the F1-score, defined as the harmonic mean of precision and recall, was used to evaluate performance. This design was intended to evaluate the comprehensive detection performance and stability of the model without relying on a single metric. It also reflects the trade-off between precision and recall. All evaluation metrics reported in the following tables are expressed as percentages.

The proposed model was implemented using PyTorch 2.4.1 (PyTorch Foundation, San Francisco, CA, USA). Experiments were conducted on Windows 11 (Microsoft Corporation, Redmond, WA, USA) using an Intel Core i7-13700K (3.40 GHz) CPU (Intel Corporation, Santa Clara, CA, USA), an NVIDIA GeForce RTX 3060 GPU (NVIDIA Corporation, Santa Clara, CA, USA), and 32 GB of RAM. Hyperparameters were finely tuned through repeated experiments to ensure optimal performance and training stability. The Adam optimizer was employed for both the generator and discriminator, with the momentum parameters set to $\beta_{1}=0.5$ and $\beta_{2}=0.999$. Differential learning rates were applied to prevent mode collapse and maintain a balance between the two networks. The learning rates for the generator and discriminator were set to $3\times 10^{-5}$ and $1\times 10^{-5}$, respectively, to prevent the discriminator from converging too rapidly. Additionally, the proportion of explicitly labeled data was set to 5% of the total dataset, and an additional 25% of the total dataset was used as the Phase 2 training subset for likelihood-based boundary refinement. The remaining data were reserved for out-of-sample evaluation. Accordingly, scores greater than 0.5 were mapped to the positive class (attack), whereas scores less than or equal to 0.5 were mapped to the negative class (normal). To quantify run-to-run stability, each leave-one-class-out SGAN scenario was independently repeated 10 times with different random initializations. Table 3 reports the resulting mean ± standard deviation values, whereas Table 4 and Table 5 report only the corresponding mean values for concise presentation.

### 5.3. Experimental Results

In this paper, a leave-one-class-out strategy was adopted to validate the generalized detection performance of the SGAN across various attack types. Specifically, one of the four attack types—DoS, fuzzy, gear, and RPM—was selected as an unknown attack and completely excluded from the training data. The model was then trained using only the remaining three attack types and normal data, and the corresponding SGAN results are reported in Table 3. For the withheld attack class, SGAN achieved accuracies of 99.67 ± 0.17%, 99.79 ± 0.04%, 99.69 ± 0.04%, and 99.78 ± 0.04% in the DoS, fuzzy, gear, and RPM scenarios, respectively. The corresponding F1-scores were 99.42 ± 0.18%, 99.69 ± 0.06%, 99.64 ± 0.05%, and 99.75 ± 0.04%, yielding an average unknown attack F1-score of 99.63%. These results indicate that the proposed framework maintains high and stable performance even when one attack type is completely excluded from training.

An experiment was conducted to determine the window size used for CAN image generation. Table 4 compares the mean performance for windows of 16, 32, 64, and 128 frames. The 16- and 32-frame settings provided insufficient temporal context and therefore showed lower detection performance. The 64-frame setting achieved the best overall results, with an average accuracy of 99.73% and an average F1-score of 99.63% across the four scenarios. Although the 128-frame setting increased the input size and computational cost, it did not improve performance over the 64-frame setting. Window size also determines the buffering delay because one decision is produced after accumulating *W* consecutive frames. If the average CAN frame arrival rate is *R* frames/s, the minimum windowing delay is W/R; relative to the 64-frame setting, the 16-, 32-, and 128-frame settings correspond to approximately 0.25×, 0.5×, and 2× the buffering delay, respectively. Therefore, 64 frames were selected as a practical trade-off between detection accuracy, computational efficiency, and windowing latency.

Based on this, the performance was analyzed by selecting the supervised learning model DCNN [10], the unsupervised learning models deep autoencoder (DAE) [31] and GIDS [19], and the semi-supervised learning model CAAE [21] as comparison groups. The results are presented in Table 5. DCNN is a supervised learning model that achieves high detection performance. In this paper, DCNN was utilized as a benchmark to assess the performance level of the proposed model. DAE and GIDS are unsupervised learning models capable of detecting untrained attacks based on reconstruction error and the normal data distribution learned through a GAN, respectively. The performance metrics for DCNN, DAE, and GIDS are available in the original papers. Unlike DAE and GIDS, CAAE is a semi-supervised learning model similar to the proposed model; however, the experimental method for defining unknown attacks is inherently different. In the original paper of CAAE, unlabeled unknown attack samples were included in the training set, allowing the model to learn the statistical manifold of the corresponding attacks in advance.

In other words, the model performs detection after experiencing attack patterns without labels. Conversely, a scenario was designed to completely exclude specific attack types during the training process. This was intended to verify the identification capability of the model for attacks that it had never encountered before. Therefore, CAAE was re-evaluated using the same leave-one-class-out procedure to reduce differences in the evaluation protocol and enable a more direct comparison. To clarify the scope of the baseline comparison, the DCNN, DAE, and GIDS values in Table 5 are reported from the original publications and are included as reference results on the same public dataset. The corresponding studies do not report fully matched training splits, hyperparameter schedules, or implementation details for the present protocol. Therefore, these numbers should be interpreted as contextual references rather than strictly controlled reproductions. By contrast, CAAE was additionally evaluated under the leave-one-class-out protocol used in this study because it is the most directly comparable semi-supervised baseline. Therefore, the most direct controlled comparison in this paper is between SGAN and CAAE, while DCNN, DAE, and GIDS are reported to situate the proposed model relative to prior literature.

As shown in Table 5, SGAN maintained favorable performance across all unknown attack scenarios. Although CAAE showed a slightly higher F1-score in the DoS attack scenario, SGAN achieved a mean F1-score of 99.69% in the fuzzy attack scenario, whereas CAAE dropped to 78.70%. SGAN also maintained mean F1-scores of 99.64% and 99.75% in the gear and RPM scenarios, respectively. Overall, the comparison indicates that the proposed model provides robust detection performance against various types of unknown attacks under the evaluated leave-one-class-out benchmark setting, while the strongest direct comparison is with the re-evaluated CAAE baseline.

The FLOPs and parameter counts in Table 6 provide a comparative view of computational efficiency in resource-constrained settings [32]. The SGAN recorded 3.25 M FLOPs, significantly reducing the computational load compared with the other models. This corresponds to approximately 3.25% of the FLOPs required by DCNN, 6.33% of those required by GIDS, and 4.23% of those required by CAAE. Furthermore, the compact parameter count of 0.21 M suggests that the proposed architecture is promising for resource-constrained embedded environments, although dedicated hardware validation remains future work.

### 5.4. Analysis and Limitations

Across the experiments, SGAN maintained high detection performance on both known and withheld attacks. The discriminator first establishes a normal–attack decision boundary from the small labeled subset and then refines the normal and abnormal regions using the Phase 2 training subset and generator-produced samples through likelihood-based objectives. This differs from GIDS and CAAE, which rely primarily on adversarially learned normal-data distributions and reconstruction errors, respectively. The proposed framework therefore uses an explicitly initialized and progressively refined boundary, together with a fixed threshold of 0.5 that is applied consistently across all scenarios without post hoc adjustment.

From a preprocessing perspective, GIDS uses one-hot CAN-ID encoding, whose input dimension grows with the number of identifiers. Although this representation is manageable for CAN 2.0A, it scales poorly to the 29-bit identifier space of CAN 2.0B. The proposed method instead maps each CAN ID to an 8-bit grayscale value, preserving the fixed 64×9 input size for both standard and extended CAN formats. This compact representation reduces input dimensionality but is lossy because multiple identifiers can share the same grayscale value, particularly in CAN 2.0B networks with sparse or clustered identifiers. Future work will investigate multi-byte encodings or learnable ID embeddings to preserve more identifier-level information without substantially increasing the input size.

Several technical limitations remain. First, the fixed 8-byte image width cannot directly represent CAN FD payloads of up to 64 bytes [33]; extending the framework will require adaptive image sizing or effective compression of the extended payload. Second, the current SGAN is a window-based detector: each decision is produced after 64 consecutive frames have been accumulated, introducing buffering delay before inference. The present results should therefore be interpreted as window-level intrusion detection rather than fully real-time frame-level detection, and a lower-latency online variant remains an important direction for future work. Third, the experiments used a single public dataset collected from one vehicle platform, so cross-dataset and cross-vehicle generalizability remains to be validated.

## 6. Conclusions

This paper presented an SGAN-based IDS for CAN intrusion detection. The proposed framework converts consecutive CAN messages into compact grayscale images and trains the SGAN through a three-phase procedure comprising discriminator initialization, boundary refinement, and generator optimization. Through ID normalization, the same preprocessing scheme supports both CAN 2.0A and CAN 2.0B while avoiding the dimensionality growth of one-hot CAN-ID encoding.

Under leave-one-class-out evaluation on the HCRL car-hacking dataset, the proposed model achieved an average accuracy of 99.73% and an average F1-score of 99.63% for unknown attacks over 10 repeated runs. These results provide a controlled improvement over the re-evaluated CAAE baseline under the same leave-one-class-out setting and compare favorably with literature-reported unsupervised baselines used as contextual references. The model required only 3.25 M FLOPs and 0.21 M parameters. These results indicate low computational complexity and suggest suitability for resource-constrained environments.

Future work will address the current windowing latency and extend the framework to CAN FD. We will also evaluate the method on additional CAN datasets and vehicle platforms through hardware-in-the-loop and real-vehicle experiments, as well as field-programmable gate array (FPGA)- and microcontroller unit (MCU)-based implementations. These efforts will support its eventual integration into a practical intrusion prevention system (IPS).

## Figures and Tables

**Table 1:** Architecture of Generator and Discriminator.

Layer (Type)	Kernel	Output Shape
Generator		
Input *z*	-	256×1×1
ConvT_1 (ConvT + ReLU)	4×1	256×4×1
ConvT_2 (ConvT + BN + ReLU)	4×1	128×8×1
ConvT_3 (ConvT + BN + ReLU)	4×2	64×16×2
ConvT_4 (ConvT + BN + ReLU)	4×2	32×32×4
ConvT_5 (ConvT + Tanh)	4×3	1×64×9
Discriminator		
Input Data	-	1×64×9
Conv2d_1 (Conv2d + ReLU)	4×3	32×32×4
Conv2d_2 (Conv2d + ReLU)	4×2	64×16×2
Conv2d_3 (Conv2d + ReLU)	4×2	128×8×1
Conv2d_4 (Conv2d + ReLU)	4×1	256×4×1
Conv2d_5 (Conv2d + Sigmoid)	4×1	1×1×1

BN: Batch Normalization.

**Table 2:** Overview of Car Hacking Dataset.

Data Type	Normal Messages	Injected Messages
Attack-free (Normal)	988,872	–
DoS Attack	3,078,250	587,521
Fuzzy Attack	3,347,013	491,847
gear Spoofing Attack	3,845,890	597,252
RPM Spoofing Attack	3,966,805	654,897

**Table 3:** Detection performance of known and unknown attacks for each scenario.

Train Set	Test Set	Accuracy	Precision	Recall	F1-Score
Fuzzy Attack gear Attack RPM Attack	DoS Attack * Fuzzy Attack gear Attack RPM Attack	99.67 ± 0.17 99.67 ± 0.20 99.77 ± 0.13 99.78 ± 0.12	99.82 ± 0.36 99.37 ± 0.12 99.73 ± 0.31 99.72 ± 0.27	99.02 ± 0.12 99.67 ± 0.02 99.74 ± 0.02 99.80 ± 0.01	99.42 ± 0.18 99.52 ± 0.30 99.73 ± 0.15 99.76 ± 0.13
DoS Attack gear Attack RPM Attack	DoS Attack Fuzzy Attack * gear Attack RPM Attack	99.45 ± 0.11 99.79 ± 0.04 99.85 ± 0.01 99.86 ± 0.02	99.64 ± 0.16 99.72 ± 0.14 99.91 ± 0.05 99.89 ± 0.06	98.52 ± 0.39 99.66 ± 0.02 99.73 ± 0.02 99.80 ± 0.01	99.08 ± 0.19 99.69 ± 0.06 99.82 ± 0.02 99.84 ± 0.02
DoS Attack Fuzzy Attack RPM Attack	DoS Attack Fuzzy Attack gear Attack * RPM Attack	99.63 ± 0.04 99.69 ± 0.04 99.69 ± 0.04 99.80 ± 0.03	99.30 ± 0.16 99.46 ± 0.16 99.77 ± 0.11 99.80 ± 0.07	99.46 ± 0.02 99.64 ± 0.02 99.51 ± 0.03 99.76 ± 0.01	99.38 ± 0.08 99.55 ± 0.07 99.64 ± 0.05 99.78 ± 0.03
DoS Attack Fuzzy Attack gear Attack	DoS Attack Fuzzy Attack gear Attack RPM Attack *	99.68 ± 0.06 99.77 ± 0.05 99.71 ± 0.08 99.78 ± 0.04	99.52 ± 0.23 99.63 ± 0.18 99.56 ± 0.19 99.84 ± 0.10	99.43 ± 0.10 99.71 ± 0.04 99.77 ± 0.03 99.67 ± 0.05	99.47 ± 0.10 99.67 ± 0.08 99.66 ± 0.09 99.75 ± 0.04

* Denotes the unknown attack class that was excluded from the training dataset.

**Table 4:** Performance comparison across different input frame sizes.

Train Set	Test Set	Frame	Accuracy	Precision	Recall	F1-Score
Fuzzy Attack gear Attack RPM Attack	DoS Attack	16 frames 32 frames 64 frames 128 frames	78.59 97.13 99.67 99.18	87.87 94.29 99.82 99.98	31.86 96.13 99.02 97.34	46.77 95.20 99.42 98.64
DoS Attack gear Attack RPM Attack	Fuzzy Attack	16 frames 32 frames 64 frames 128 frames	93.70 92.67 99.79 96.47	93.12 84.40 99.72 99.75	87.61 95.95 99.66 89.94	90.28 89.80 99.69 94.59
DoS Attack Fuzzy Attack RPM Attack	gear Attack	16 frames 32 frames 64 frames 128 frames	96.02 98.97 99.69 99.55	97.06 98.14 99.77 99.96	93.46 99.46 99.51 99.01	95.22 98.80 99.64 99.48
DoS Attack Fuzzy Attack gear Attack	RPM Attack	16 frames 32 frames 64 frames 128 frames	96.98 99.32 99.78 99.78	97.14 98.69 99.84 99.99	96.08 99.82 99.67 99.52	96.61 99.25 99.75 99.76

**Table 5:** Comparison with literature-reported DCNN, DAE, and GIDS results and re-evaluated CAAE results. The DCNN, DAE, and GIDS values are taken from the original papers; CAAE was re-evaluated using the leave-one-class-out protocol of this study; and SGAN values are reported as means over 10 runs.

Train Set	Test Set	Model	Accuracy	Precision	Recall	F1-Score
Fuzzy Attack gear Attack RPM Attack	DoS Attack	DCNN ^S^ [10] DAE ^U^ [31] GIDS ^U^ [19] CAAE ^SS^ [21] SGAN ^SS^	99.97 96.24 97.9 99.40 99.67	100 91.27 96.8 99.90 99.82	99.89 99.88 99.6 99.22 99.02	99.95 95.38 98.2 99.56 99.42
DoS Attack gear Attack RPM Attack	Fuzzy Attack	DCNN ^S^ [10] DAE ^U^ [31] GIDS ^U^ [19] CAAE ^SS^ [21] SGAN ^SS^	99.82 93.87 98.0 77.97 99.79	99.95 90.05 97.3 99.93 99.72	99.65 96.26 99.5 64.91 99.66	99.80 93.05 98.4 78.70 99.69
DoS Attack Fuzzy Attack RPM Attack	gear Attack	DCNN ^S^ [10] DAE ^U^ [31] GIDS ^U^ [19] CAAE ^SS^ [21] SGAN ^SS^	99.95 88.02 96.2 99.62 99.69	99.99 94.63 98.1 99.78 99.77	99.89 81.80 96.5 98.92 99.51	99.94 87.75 97.3 99.35 99.64
DoS Attack Fuzzy Attack gear Attack	RPM Attack	DCNN ^S^ [10] DAE ^U^ [31] GIDS ^U^ [19] CAAE ^SS^ [21] SGAN ^SS^	99.97 91.60 98.0 99.44 99.78	99.99 95.73 98.3 99.83 99.84	99.94 95.73 99.0 98.13 99.67	99.96 92.10 98.65 98.97 99.75

^S^ Supervised, ^U^ Unsupervised, ^SS^ Semi-supervised.

**Table 6:** Comparison of computational complexity and model size.

Model	Methodology	FLOPs	Parameters
DCNN [10]	Supervised	100.13 M	1.71 M
GIDS [19]	Unsupervised	51.32 M	0.13 M
CAAE [21]	Semi-supervised	76.78 M	2.15 M
SGAN	Semi-supervised	3.25 M	0.21 M

## Data Availability

Publicly available data were analyzed in this study. These data can be found at the HCRL Car-Hacking Dataset: https://ocslab.hksecurity.net/Datasets/car-hacking-dataset/ (accessed on 13 April 2026).
